# Supplementary material for: Causal Relationship between Aging and Anorexia Nervosa: A White-Matter-Microstructure-Mediated Mendelian Randomization Analysis
Source: Biomedicines. 2024 Aug 16;12(8):1874. doi: 10.3390/biomedicines12081874 (PMC11351342; doi:10.3390/biomedicines12081874)
Supplement: Supplementary file 1 [file biomedicines-12-01874-s001.zip › Supplementary Figure/Figure S8/Leaveoneout plot of MR analysis for TL to ubm-b-1740.pdf]

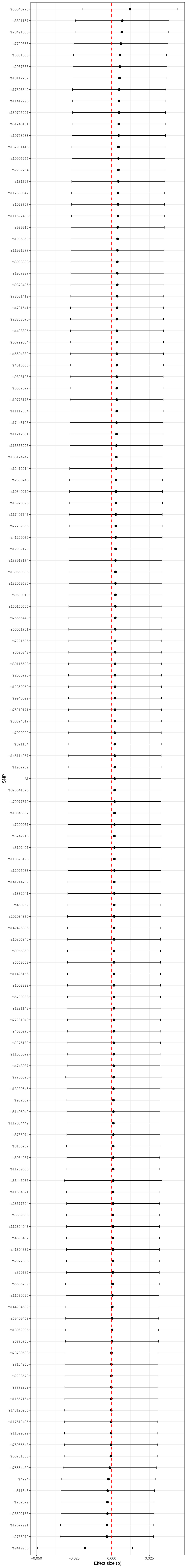
rs35640778
rs3891167
rs78491606
rs7790856
rs6881568
rs2967355
rs10112752
rs17803849
rs11412296
rs139795227
rs61748181
rs10768683
rs137901416
rs10905255
rs2282764
rs131797
rs117630647
rs1023767
rs111527438
rs939916
rs1985369
rs11991877
rs3093888
rs1957937
rs9878436
rs73581419
rs4731541
rs28363070
rs4498805
rs56799554
rs45604339
rs4616688
rs9398196
rs6587577
rs10773176
rs11117354
rs17445108
rs11212631
rs116863223
rs185174247
rs12412214
rs2538745
rs10840270
rs16978028
rs117407747
rs77732866
rs41269079
rs12932179
rs188918174
rs139669835
rs182059586
rs9600019
rs150150565
rs76666449
rs56061761
rs7221585
rs6590343
rs80116508
rs2056726
rs12369950
rs9940099
rs76219171
rs80324517
rs7099229
rs871134
rs145114957
rs1907702
All
rs376641875
rs79977579
rs10845387
rs7209057
rs5742915
rs8102497
rs113525195
rs12925933
rs141214782
rs1332941
rs450962
rs202034370
rs142426306
rs10805346
rs9955360
rs6659669
rs11426156
rs1003322
rs6790988
rs1291143
rs77231040
rs4530278
rs2276182
rs11085072
rs4743037
rs7705526
rs13230646
rs932002
rs61405042
rs117034449
rs3785074
rs8105767
rs6054257
rs11769630
rs35446936
rs11584821
rs28577594
rs6669563
rs112394943
rs4695407
rs41304832
rs2977608
rs869785
rs6536702
rs11579626
rs144204502
rs59409453
rs13062095
rs6776756
rs73730598
rs7164950
rs2293579
rs7772289
rs11557154
rs143190905
rs117512405
rs11699829
rs76065543
rs66731853
rs75664430
rs4724
rs611646
rs762679
rs28502153
rs17677991
rs2763979
rs9419958
SNP
−0.050
−0.025
0.000
0.025
Effect size (b)
